# Economic analysis using higher-frequency time series: challenges for seasonal adjustment

**DOI:** 10.1007/s00181-022-02287-5

**Published:** 2022-08-06

**Authors:** Daniel Ollech, Deutsche Bundesbank

**Affiliations:** Central Office, Directorate General Statistics, Wilhelm-Epstein-Strasse 14, 60431 Frankfurt am Main, Germany

**Keywords:** COVID-19, DSA, Calendar adjustment, Time series characteristics, C14, C22, C87, E66

## Abstract

The COVID-19 pandemic has increased the need for timely and granular information to assess the state of the economy in real time. Weekly and daily indices have been constructed using higher-frequency data to address this need. Yet the seasonal and calendar adjustment of the underlying time series is challenging. Here, we analyse the features and idiosyncracies of such time series relevant in the context of seasonal adjustment. Drawing on a set of time series for Germany—namely hourly electricity consumption, the daily truck toll mileage, and weekly Google Trends data—used in many countries to assess economic development during the pandemic, we discuss obstacles, difficulties, and adjustment options. Furthermore, we develop a taxonomy of the central features of seasonal higher-frequency time series.

## Motivation

During the COVID-19 pandemic, policymakers and economists sought more timely and granular information on the state of the economy. To this end, higher-frequency indices that track the economic development of a country have been developed. Most prominently, Lewis et al. (2020) developed the Weekly Economic Index (WEI) for the USA that combines several weekly indicators such as US railroad traffic, electric utility output or unemployment insurance claims. The WEI tracks the output of the US economy and allows the state of the economy to be monitored on a weekly basis (Lewis et al. 2020, 2021).

The WEI has sparked the development of a series of similar weekly indicators, especially for European countries (e.g. Delle Monache et al. 2020; Eraslan and Götz 2020; Fenz and Stix 2021; Wegmüller et al. 2021). Lourenço and Rua (2021) develop a daily variant (DEI) for the Portuguese economy. Woloszko (2020) constructs country-specific economic indicators for 46 countries based on Google Trends data.

During the last decade, the number of seasonal adjustment methods for higher-frequency time series has seen a considerable increase (for a discussion and further references see, De Livera et al. 2011; Ladiray et al. 2018; Ollech 2021; Proietti and Pedregal 2022; Webel 2020). Yet, in the construction of the WEI, DEI and some of the other indices, these methods are not employed. Instead, ad-hoc measures like year-on-year rates are used to handle the seasonality of the data. This does not usually take into account calendar effects or the structure of the periodic and seasonal effects such as the varying number of weeks per year.

The higher-frequency time series used in the construction of the economic indices often present methodological challenges that differ substantially from those encountered in lower-frequency data. In this paper, we therefore contribute to the literature by analysing and seasonally adjusting a set of higher-frequency time series that is typical of the kind of data relied upon by economists and business cycle analysts during the COVID-19 pandemic. As the literature on the adjustment and modelling of higher-frequency seasonal time series is growing rapidly, the lack of a common understanding of the features, that need to be taken into account when modelling and adjusting these data, becomes evident. Therefore, another key contribution of the analysis presented here is the derivation and systematization of those characteristics that are relevant in the context of seasonal adjustment.

To this end, Sect. 2 discusses the characteristic features of higher-frequency time series from a seasonal adjustment perspective. Section 3 presents the seasonal adjustment methods employed in this study, while Sect. 4 analyses and adjusts the daily truck toll mileage, the hourly electricity consumption and a weekly Google Trends series. These series are used as inputs to the Weekly Activity Index (WAI) devised by Eraslan and Götz (2020). Section 5 summarizes.

**Table 1:** Key notions for the adjustment of higher-frequency data

Notion	Description
*Basic characteristics*	
Many observations	Higher-frequency data contain many observations which can be a challenge for algorithms and users.
Short series	Many series contain few years of observations.
Temporal aggregation	Feasible temporal aggregation of adjusted series depend on data type.
Non-isochronicity	Number of observations per periodic cycle is not always the same for all cycles, e.g. number of weeks per year.
Non-equidistance	For some series, the distance between observations varies, e.g. bank-daily, with 0 to $3+$ days between observations.
Date and time conventions	Conventions regarding the start of the week or year Numbering impact the data structure.
*Periodic and calendar effects*	
Multiple periodic effects	Daily time series usually contain day-of-the-week and day-of-the-year effects.
Multilevel periodic effects	Seasonal structure may be hierarchical, e.g. a series with hour-of-the-day and day-of-the-week effects.
Uncommon periodic effects	Higher-frequency series may contain other periodic effects, such as week-of-the-month effects.
Breaks in periodic effects	Periodic effects may change rapidly, e.g. as a consequence of fundamental changes in the data generating process.
Cross-seasonality	The periodic and calendar effects can be interdependent.
Autocorrelational Seasonality	The seasonal impact of consecutive observations may be highly dependent.
Series-specific calendar effects	Calendar effects can be observed more directly, so regressor construction can more easily be tailored to the series.
Uncommon calendar effects	Higher-frequency series may contain other calendar effects, such as daylight saving time.
Bridge days	Bridge days may have a traceable effect on the series.
*Outliers and missing values*	
Missing values	Due to data availability, some series contain (temporarily) missing values, often at the end of the series.
Unreliable data delivery	Data producers do not necessarily have an obligation to deliver data or provide additional information.
Higher volatility	The volatility of the time series usually decreases with a higher temporal aggregation.
Heteroskedasticity	The volatility may change over time and may be seasonal.
Non-traditional outlier patterns	Observed outlier pattern can be different from lower frequency series, e.g. slower rate of decay in a TC outlire.

## Key notions for the adjustment of higher-frequency data

Seasonal higher-frequency time series show features and idiosyncracies that are of immediate importance for any seasonal adjustment procedure. The time series examples discussed in the next sections include some or all of the characteristics summarized in Table 1. To guide the readers understanding and as a point of reference, this table is included at the beginning of this paper.

Table 1 organizes the characteristics of seasonal higher-frequency time series into a taxonomy of relevant features.

The basic characteristics cover the most obvious features that concern the whole set of observations. The fact that higher-frequency time series contain many observations—especially in comparison with lower-frequency time series—is in itself trivial. Yet, it increases the computational burden and may render some methods inapplicable. Non-isochronicity and non-equidistance will usually either have to be addressed by adapting the methods used or by transforming the data, e.g. by interpolation or time-warping.

The periodic and calendar effects encompass concepts that extend the definition of seasonality and calendar effects in lower-frequency time series. As becomes clear from the examples of daily data, higher-frequency time series often contain multiple noticeable periodic effects that may be interdependent and autocorrelational. For calendar effects, we may consider including constellations that are not recommended to adjust for in lower-frequency time series, such as bridge days. This may be sensible as the higher number of observations allow us to observe and estimate such effects more directly. For weekly series, the impact of bridge days may even be inseparable from the moving holidays, if they always fall in the same week.

Higher-frequency time series are generally more volatile than traditional business-cycle indicators. This is mostly a result of the fact that irregular influences tend to partially offset each other over a longer time span and are therefore less pronounced in monthly and quarterly data. Additionally, many of the higher-frequency time series encountered are merely a by-product and are not compiled for economic analysis or official statistics and therefore do not adhere to the same data standards. Accordingly, the methods applied to higher-frequency time series usually need to be robust against outliers and irregular observations.

## Seasonal adjustment methods

The focus of this paper lies on the qualitative assessment of higher-frequency time series and to this end, we employ methods for the seasonal adjustment of such series to guide our understanding. Although we discuss the seasonal adjustment results and highlight potential challenges, we do not advocate for any particular method. Instead, the findings shall carve out features of higher-frequency time series that need to be addressed regardless of the method applied.

Let $\{Y_t\}$ denote a series of length *T* with cycle length $\tau$. For monthly series, $\tau$ equals the number of observations per year, i.e. 12. For a daily series with only a weekday pattern, the recurring pattern has a cycle length of 7. The basic time series decomposition is then given by

1 $$\begin{aligned} Y_t = T_t + S_t + C_t + I_t \end{aligned}$$

which includes the trend-cycle ($T_t$), seasonal ($S_t$), calendar ($C_t$) and irregular component ($I_t$). It can easily be adapted to capture a multiplicative relationship between the components by log-transformation of the original time series.^1^ For multiple periodic effects with cycle lengths $\tau _1, \tau _2, ...$, the basic time series model can be generalized to

2 $$\begin{aligned} Y_t = T_t + \sum _{i} S_t^{(\tau _i)} + C_t + I_t \end{aligned}$$

### Remark 1

In Eq. 2, the periodic effects are subsumed as seasonal effects to allow a parsimonious notation. Here, effects related to calendar constellation are not considered to be periodic effects, even though the calendar constellation recurs with a cycle length of 400 years in the Gregorian calendar. However, this recurrence is not exploited in the modelling of the time series and effects arising from these calendar constellations will not usually recur in similar intensity every 400 years.

As we will see, for some series, different seasonal effects are interdependent. Likewise, calender effects and seasonal effects can be related. If all such interactions are relevant, Eq. 2 needs to be augmented so that

3 $$\begin{aligned} Y_t = T_t + \sum _{i} S_t^{\tau _i} + C_t + \sum _{i} \sum _{j > i} S_t^{(\tau _i)}*S_t^{(\tau _{j})} + \sum _{i} S_t^{\tau _i}*C_t + I_t. \end{aligned}$$

In line with the methods used for official seasonal adjustment—namely X-13 and Tramo-Seats—the adjustment of the higher-frequency time series presented here combines a seasonal adjustment routine with a RegARIMA-based pre-adjustment. The latter is a regression model (Reg) with autoregressive integrated moving average (ARIMA) errors and is used here for the estimation and elimination of calendar and some of the interaction effects. The RegARIMA model is given by

4 $$\begin{aligned} \phi _p(B) \phi _P(B^{\tau })(1-B)^d(1-B^{\tau })^D \left( Y_t - \sum _{i=1}^r \beta _i X_{it} \right) = \theta _q (B) \theta _Q(B^{\tau }) \varepsilon _t \end{aligned}$$

where $\phi (B)$ and $\theta (B)$ are AR and MA polynomials of order *p* and *q*, number of differences *d*, and capitals indicating seasonal terms while *B* is the backshift operator, i.e. $B(y_t)=y_{t-1}$. The parameter $\beta _i$ captures the impact of the *i*th regressor $X_{it}$ on the time series $Y_t$ and $\varepsilon _t$ is the error term. The ARIMA part of Eq. 4 can be abbreviated by $(p \, d \, q)(P \, D \, Q)_{\tau }$. Extensions of this model to multiple seasonalities are known and available (e.g. Svetunkov 2017), but at the time of writing, these are rarely used in seasonal and calendar adjustment.

### Seasonal adjustment of daily time series

The iterative daily seasonal adjustment (DSA) procedure described by Ollech (2018; 2021) combines the aforementioned RegARIMA model with STL (Seasonal and Trend decomposition using Loess, Cleveland et al. 1990).

DSA can integrate other seasonal adjustment methods as well, if they are flexible enough to handle daily data. In this regard, Ollech et al. (2021) discuss the flexibilization of X-11 to handle higher-frequency time series.

STL decomposes a time series into $T_t$, $S_t$ and $I_t$ using a series of Loess regressions and moving averages to separate out the trend and a periodic pattern from the series. Loess regressions are locally weighted linear or polynomial regressions (Cleveland and Devlin 1988). Each observation is regressed on a pre-defined set containing the $\gamma _\tau$ closest observations. These observations are weighted, where the weight depends negatively on the distance between observations as follows: With a reference observation—i.e. the observation to be regressed on its neighbouring observations—at time *t*, the weight of the observation at $t_i$ is given by

5 $$\begin{aligned} v_{i}(t)=\left[ 1-\left( \frac{|t_{i}-t|}{\delta _{\gamma _\tau }(t)}\right) ^{3}\right] ^{3} \end{aligned}$$

where $\delta _{\gamma _\tau }(t)=|t_{i}-t_{\gamma _\tau }|$ is the distance between the $\gamma _\tau ^{th}$ farthest $t_i$ and *t*.

In the robust version of STL, after the decomposition of $Y_t$ into a trend, seasonal and irregular component (inner loop) an extreme value downweighing is added (outer loop). Observations with extreme values get a weight $\omega _t$ in the local regressions in the next iterations given by

6 $$\begin{aligned} \omega _t = {\left\{ \begin{array}{ll} \left( 1-\left[ \frac{ |I_t| }{6 \cdot median(|\{I_t\}_{t=1}^N|)}\right] ^2\right) ^2 &{} \text {if} \; \; |I_t| < 6 \cdot median(|\{I_t\}_{t=1}^N|) \\ 0 &{} \text {else} \end{array}\right. } \end{aligned}$$

The inner loop iterates through the following steps:

1. Trend adjustment: $$Y_t-T_t^{\{k-1\}} = S_t^{\{k\}} + I_t^{\{k\}} \equiv TA _t^{\{k\}}, \; \text {with} \ T_t^{\{0\}}={\textbf {0}}$$.
2. Preliminary periodwise smoothing: Each periodwise subseries of $TA _t^{\{k\}}$ is smoothed by Loess to yield a preliminary seasonal factor $S_{pre, t}^{\{k\}}$. The $\gamma _\tau$ has to be specified by the user as there are no default values available (see, Cleveland et al. 1990).
3. Smoothing preliminary seasonal component to capture any low-frequency movements $L_t^{\{k\}}$ from $S_{pre, t}^{\{k\}}$.
4. Obtaining seasonal component: $S_t^{\{k\}}=S_{pre, t}^{\{k\}}-L_t^{\{k\}}$.
5. Seasonally adjusting the original time series: $Y_t^{\{k\}}-S_t^{\{k\}} \equiv SA _t^{\{k\}}$.
6. Obtaining trend: Apply a Loess filter to $SA _t^{\{k\}}$ to extract $T_t^{\{k\}}$.

STL only extracts one periodic pattern at a time. Therefore, DSA combines multiple runs of STL with RegARIMA:

- Step I: Adjust intra-weekly seasonality with STL.
- Step II: Calendar- and outlier adjustment with RegARIMA.
- Step III: Adjust intra-monthly seasonality with STL.
- Step IV: Adjust intra-annual seasonality with STL.

In the intermediate steps, the remaining periodic effects are included in the trend-cycle component, which by default in STL captures low-frequency variation that is lower than the considered seasonal frequency.^2^

The resulting final seasonally adjusted series will be adjusted for all seasonal and moving holiday effects considered. The order of the steps in DSA follows the maxim to start with the periodic pattern with the shortest cycle length, i.e. the intra-weekly seasonality. Unlike the methods used for seasonal adjustment of lower-frequency time series, the RegARIMA part is not the first step, as it would necessitate to estimate a RegARIMA model with multiple seasonal parts. Future versions of DSA may include such an extension.

For all computations and analyses, we use R 4.0.2 (R Core Team 2021). The seasonal adjustment of daily time series is computed using the {dsa} package in version 1.1.15.

### Seasonal adjustment of hourly time series

Hourly series are challenging due to the large number of observations included. For computational reasons, it will often not be possible to estimate a RegARIMA model for such series, if many regressors need to be included. Here we adapt the DSA procedure to the case of hourly data as follows:

- Step I: Adjust hour-of-the-week effects with STL.
- Step II: Calendar- and outlier adjustment with RegARIMA based on daily observations.
- Step III: Adjust hour-of-the-month effects with STL.
- Step IV: Adjust hour-of-the-year effects with STL.

As with DSA, single steps of the routine used to seasonally adjust hourly data may be omitted, e.g. only a few series will exhibit an hour-of-the-month effects. Also, the first step may be changed to model hour-of-the-day effects instead or in addition to day-of-the-week effects (see Remark 9).

#### Remark 2

The transformation from hourly to daily observations in step II only serves to reduce the computational burden. For some series, it may be feasible and preferable to directly estimate the calendar effects in the hourly series—provided an hourly RegARIMA model can be computed. If a distinct moving holiday impact for each hour of a holiday is assumed, the number of regressors may be extremely large. This is aggravated if cross-seasonal effects need to be modelled, e.g. if interactions between fixed holidays, the weekday and potentially the hour are prevalent.

### Seasonal adjustment of weekly time series

On average, a year contains a non-integer number of weeks, namely 52.18. Ladiray et al. (2018) describe how, in these cases, autoregressive fractionally integrated moving average (ARFIMA) processes can be exploited that adapt the seasonal differencing part of Eq. 4 to incorporate fractionally integrated processes. The authors develop a fractional variant of the well-known airline model, i.e. the seasonal ARIMA model of order $(0,1,1)(0,1,1)_\tau$. For a non-integer seasonal period of $\tau =\lfloor \tau \rfloor +\alpha$, with $\alpha \in [0,1]$, the fractional differencing operator $\tilde{\nabla }_{\tau }$ can be approximated by a first-order Taylor series expansion so that

7 $$\begin{aligned} \tilde{\nabla }_{\tau }Y_t \approx Y_t - (1-\alpha ) B^{\lfloor \tau \rfloor } Y_t - \alpha B^{\lfloor \tau \rfloor +1} Y_t \end{aligned}$$

The fractional airline model can be used for linearization of a time series analogously to Tramo.^3^ We combine this pre-processing with a SEATS-type time series decomposition of the fractional airline model to seasonally adjust weekly time series with $\tau =52.18$.

## Empirical illustrations

We will present a small set of higher-frequency time series that have been of high importance for economic analysis during the COVID-19 pandemic. We will discuss key features of this series and show how these might be accounted for when conducting calendar and seasonal adjustment.

### Truck toll mileage index

**Fig. 1:**
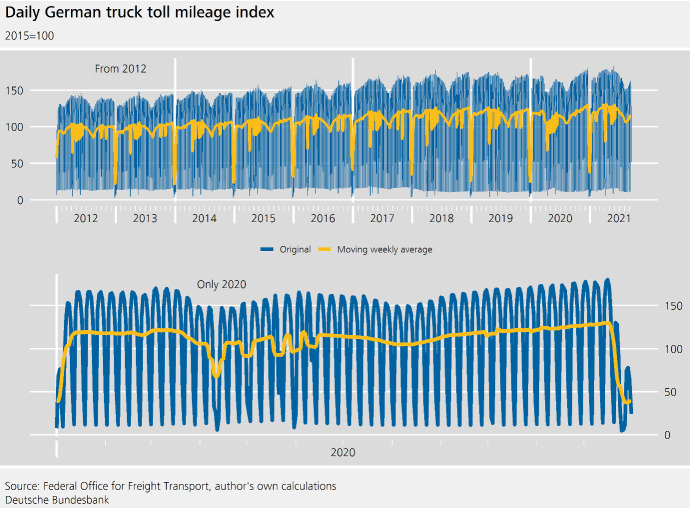
Daily German truck toll mileage index

The Federal Office for Freight Transport in Germany is responsible for a distance-based toll on trucks, which was implemented in January 2005. The truck toll mileage index has been developed together with the Federal Statistical Office. It is based on raw mileage and free of structural breaks resulting from changes in the vehicles that have to pay the toll. The data are available as a monthly and a daily index (Deutsche Bundesbank, 2020). The daily time series analysed here is available from 1 January 2005 up to 12 September 2021, and thus contains **many observations**.

As can be seen from Fig. 1, the series is characterized by **multiple periodic effects**, namely a strong weekday pattern, with a trough on Sundays and an annual pattern. These effects are interdependent: the **cross-seasonality** presents itself as a weekday pattern that changes throughout the year. In part, this is due to governing laws and regulations: with only a few exceptions, trucks are not permitted to drive on motorways on Sundays or on public holidays. During July and August, this is extended to Saturdays as well. We further observe different patterns around Christmas related to different changes in consumption behaviour.

#### Remark 3

Other series contain less typical or even **uncommon periodic effects**. Ollech (2021) finds a monthly recurring pattern in currency in circulation in Germany.

The series is further marked by a weakly positively sloping trend that is halted temporarily in early 2020, as a consequence of the COVID-19 pandemic. After the COVID-19-induced slowdown, we observe **breaks in periodic effects**, in particular the weekday pattern. At least temporarily, the difference in the truck mileage between workdays and weekend days is less pronounced. The annual seasonal pattern is **non-isochronous**, i.e. the number of observations per cycle is not the same for all cycles as years contain either 365 or 366 observations.

#### Remark 4

Non-isochronicity can impact a time series in a number of ways. We may observe that the seasonal pattern in longer cycles is just a stretched-out version of the pattern in shorter cycles. This might be the case, if the seasonal pattern is a smooth pattern in the sense that the seasonal impact of neighbouring values are highly correlated. In series with a more fluctuating pattern, the seasonal impact of additional observations in a given cycle, such as February 29 in a leap year, may be less related to adjacent observations. This may imply that the estimation of the seasonal impact of these additional observations in longer cycles cannot be inferred from observations in the shorter cycle.

**Fig. 2:**
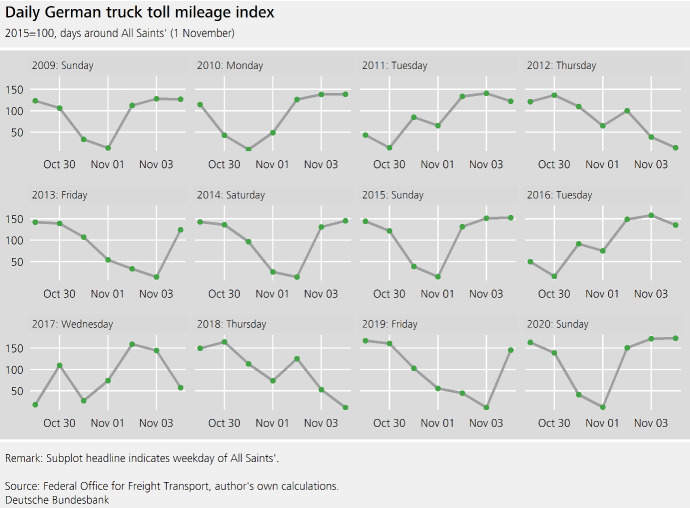
Daily German truck toll mileage index, days around All Saints’ (1 November)

For higher-frequency time series, it is often possible to observe the impact of holidays more directly, and thus identify **series-specific calendar effects**. Figure 2 shows the truck toll mileage index on All Saints’ Day (November 1) as well as the three days leading up to and the three days after the holiday. All Saints’ Day is a public holiday in 5 out of 16 German federal states. In these states, all of which are located either in the south or west of the country, trucks are prohibited from driving on motorways on this day. The impact of All Saints’ Day is cross-seasonal: the magnitude of the decrease depends on the weekday.^4^ As restrictions already apply to the driving of trucks on Sundays, All Saints’ Day does not have an additional impact on the truck toll mileage index if it falls on a Sunday. By contrast, there is a considerable reduction if it falls on any given weekday.

If All Saints’ Day falls on a Tuesday (Thursday), the neighbouring Monday (Friday), i.e. any **bridge days**, also appear to have a reduced number of trucks on motorways. In contrast to lower-frequency time series, these bridge day effects can be seen and modelled more directly.

The COVID-19 pandemic, which hit Germany in March 2020, has a twofold impact on the time series. First, it leads to an abrupt decline in the number of kilometres driven by trucks. Second, it impacts the observed weekday pattern owing to changes in the restrictions regarding on which days trucks are allowed to drive on motorways and the consumption pattern.

To obtain a calendar and seasonally adjusted series, DSA was used with $\gamma _7:=7$ and $\gamma _{365}:=11$ using an additive decomposition. For the seasonal adjustment of the monthly index, we found a multiplicative decomposition to be more appropriate. Yet due to the **higher volatility** of the daily index—which is typical for higher-frequency time series—the seasonal factors from a multiplicative model would inflate strong outliers in many cases, leading to extreme spikes in the seasonally adjusted series. The series does not contain day-of-the-month effects^5^; accordingly, step III of the DSA procedure is omitted. The choice of a very short filter to estimate the day-of-the-week effect, i.e. $\gamma _7:=7$, allows the day-of-the-week-effect to change during the year, thus making it possible to capture this particular cross-seasonal effect. As discussed above, this is important, because we already know that the weekday pattern is different in different parts of the year, due to the aforementioned driving restrictions. The day-of-the-year effect is captured using $\gamma _{365}:=11$. This is appropriate because it allows the seasonal effect to change slowly over time, but does not overreact to single years. An important tool to identify an appropriate value for $\gamma _\tau$ are visualizations of the seasonal-irregular component (Cleveland and Terpenning 1982), so-called SI-ratios. These are frequently used to visualize the volatility of the combined seasonal-irregular series and a plausible trajectory of the seasonal component.

**Fig. 3:**
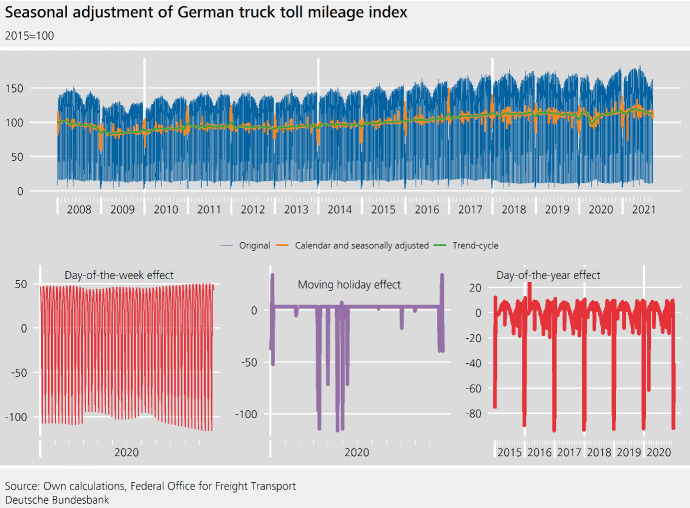
Seasonal adjustment of German truck toll mileage index

To estimate the impact of moving holiday effects and the interaction between weekdays and fixed holidays, a RegARIMA model is used. As described by Ollech (2021), this model combines a non-seasonal ARIMA model with trigonometric terms that capture deterministic seasonality. For the truck toll mileage index, 30 cosine and sine terms are used. Ollech (2021) states that multiples of 12 capture intra-monthly pattern, yet here, the high number of trigonometric terms used instead reflects the complexity of the seasonal pattern and does not indicate a day-of-the-month effect.

**Table 2:** Estimated moving holiday and cross-seasonal effects for the German truck toll mileage index

Estimate		S.E.	Estimate		S.E.
Carnival Monday	$-8.6$	1.6	NH$^1$ (Mon)	$-92.5$	2.3
Mardi Gras	$-5.0$	1.6	NH$^1$ (Tue)	$-106.3$	2.4
Holy Thursday	$-20.2$	1.5	NH$^1$ (Wed, to 2015)	$-90.4$	3.1
Good Friday	$-99.7$	1.9	NH$^1$ (Wed, from 2015)	$-118.8$	2.7
Holy Saturday	$-17.3$	1.9	NH$^1$ (Thu)	$-106.7$	2.1
Easter Sunday	$-10.4$	1.9	NH$^1$ (Fri)	$-74.6$	1.9
Easter Monday	$-117.6$	1.9	NH$^1$ (Sat)	$-20.6$	1.9
Easter Monday (t+1)	$-21.4$	1.5	Christmas Period (Mon)	$-32.7$	1.6
Ascension (t–1)	$-13.4$	1.5	Christmas Period (Tue)	$-42.5$	1.7
Ascension (to 2015)	$-109.4$	2.2	Christmas Period (Wed)	$-41.7$	1.7
Ascension (from 2016)	$-119.4$	2.3	Christmas Period (Thu)	$-41.4$	1.7
Ascension (t+1)	$-18.4$	1.6	Christmas Period (Fri)	$-28.7$	1.7
Corpus Christi (t–1, to 2015)	$-7.4$	2.0	3d before Christmas (Sun)	22.8	2.1
Corpus Christi (to 2015)	$-73.8$	2.2	Christmas Eve (Sat)	11.7	4.3
Corpus Christi (from 2016)	$-74.5$	2.1	Christmas Eve (Sun)	42.0	4.8
Corpus Christi (t+1)	$-7.7$	1.5	Christmas Day (Sat)	24.9	5.9
Pentecost (t-1)	4.1	1.3	Christmas Day (Sun)	58.4	4.3
Pentecost (to 2015)	$-107.7$	1.9	Boxing Day (Sat)	18.7	3.2
Pentecost (from 2016)	$-117.6$	2.1	Boxing Day (Sun)	30.4	5.8
Pentecost (t+1)	$-14.2$	1.5	10d after Dec 26 (Sat)	14.8	1.8
Labour Day (bridge)	$-17.9$	2.6	10d after Dec 26 (Sun)	30.9	1.9
German Unity (bridge)	$-2.6$	2.9			
All Saints’ Day (bridge)	$-20.6$	2.4			

Based on time series only adjusted for intra-weekly seasonal effects. A RegARIMA(2,1,1) model with $2\times 30$ trigonometric terms has been estimated

$^1$ NH includes the following holidays with fixed dates: Epiphany, Labour Day, Assumption Day, German Unity, Reformation Day and All Saints’ Day. The weights of the regional holidays are given by: Epiphany 0.2, Assumption Day 0.1, Reformation Day (after 2017) 0.2 and All Saints’ Day 0.6

Note: Ascension and Labour Day 2008 both fell on 1 May. Because the effect is not additive,

the effect has been assigned to Labour Day only, i.e. the regressor for Ascension is 0 on that day.

#### Remark 5

To obtain the unadjusted monthly truck toll mileage index, the daily raw truck toll mileage is summed up for each month and transformed into an index. If a **temporal aggregation** is likewise performed on the seasonally adjusted truck toll mileage index via summation, the resulting time series will contain a length-of-the-month effect—i.e. months with more days will have a higher value—and thus be seasonal. Using the average monthly index instead is a simple remedy, but reduces the comparability of the directly adjusted monthly and daily time series.

The calendar and seasonally adjusted truck toll mileage index is especially volatile around holidays (see Fig. 3). This **heteroskedasticity** is due to estimation uncertainty and the fact that truck drivers are restricted by laws with regard to the number of hours that they are allowed to drive per week and per day. The latter determines the optimal logistics and thus the interaction between holidays, consumer demands and kilometres driven by trucks on a given day resulting in the observed local volatility increases.

Table 2 shows the estimated impact of moving holidays and cross-seasonal effects. All moving holidays that are public holidays at the national or federal state level are included in the regression. This is extended to the surrounding days, as a transition phase can usually be observed, so that both the day before and after a given public holiday show a decrease. The only exception is the Sunday just before Pentecost Monday, which is slightly positive compared to a typical Sunday in spring. Additionally, we include the main event days of the carnival season for which a significant impact can be detected.

The impact of fixed holidays is usually captured by STL in the last step of DSA. Yet, as discussed above, the interdependency between that impact and the weekday cannot be captured by STL. Here, we model it using interaction dummies in the RegARIMA model. The impact of each weekday is combined across all national and federal state-level holidays to increase the parsimony of the estimated model. Days that are public holidays only regionally are weighted accordingly (for details on the weights see Table 2 and Deutsche Bundesbank 2020). The remaining spikes around Christmas are not seasonal, as they do not fall on the same day of each year. Yet, with more observations it may be possible to further improve the estimation of cross-seasonal effects for that time period.

The changes in the weekday pattern after the start of the COVID-19 pandemic in Germany discussed above recur weekly and may thus be considered to be part of the $S^{(7)}$-component. A different view is that as these changes are only temporary and a result of the irregular nature of the crisis they should be captured in the irregular component and thus be visible in the calendar and seasonally adjusted series.^6^ For the period from 23 March to 30 August 2020, i.e. from the beginning of the lockdown until after the summer holidays, we chose the latter approach. To seasonally adjust this period, forecasted calendar and seasonal factors are used, which are obtained by restricting the estimation span from the beginning of the time series to 22 March 2020. For the period after 30 August, we again use all available data in a controlled current adjustment scheme. This means that the seasonal and calendar components are re-estimated monthly, but we control weekly, whether a re-estimation is necessary. This is the case when the forecasted seasonal or calendar component no longer adequately capture the respective effect, e.g. because the seasonal pattern has evolved differently than predicted.

#### Remark 6

Generally, at the beginning of a crisis, it may be a good policy not to re-estimate the seasonal and calendar components immediately, in order to avoid including transitory and irregular influences into the seasonal or calendar components. Once the crisis has stabilized, a controlled current adjustment scheme as discussed above may be implemented.

### Electricity consumption

The German electricity consumption is compiled by the German Federal Network Agency using data from the network providers starting in January 2015. The series analysed here ends on 30 April 2021. Energy consumption is related to industrial production and GDP. Electricity consumption is therefore of particular interest to business-cycle analysis (Arora and Shi 2016; Do et al. 2016). For the most recent observations, the series is subject to **unreliable data delivery** as some network providers do not always provide data immediately.

#### Remark 7

More broadly, unreliable data delivery stems from the issue that data providers are often not legally or contractually obligated to provide data and do not need to adhere to any standards regarding data quality or deadlines. Furthermore, if the data are merely a by-product, it may be difficult to analyse the quality of the data.

In the case of electricity consumption, this leads to temporarily **missing values** that might require interpolation, either before or as part of the seasonal and calendar adjustment.

#### Remark 8

Some higher-frequency time series do have structurally missing values, e.g. time series that only contain observations on working days. If this is the case, the data may be **non-equidistant**, i.e. the distance in time between observations is not the same for all neighbouring observations. In other words, the distance between a Monday and a Tuesday is less than between a Friday and a Monday.

**Fig. 4:**
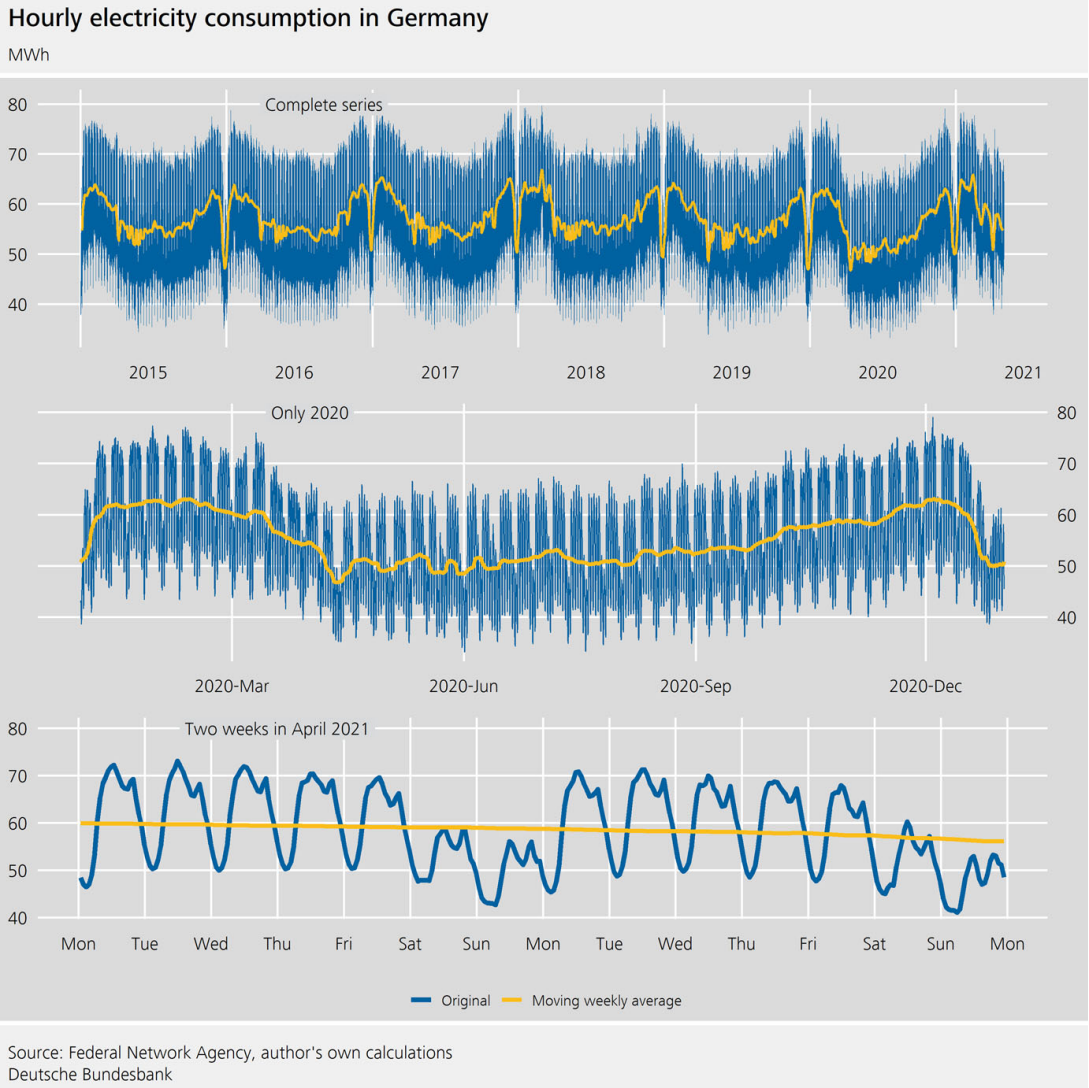
Hourly electricity consumption in Germany

Electricity consumption is available on a 15-minute basis. Ollech (2021) discusses how the daily electricity consumption can be seasonally adjusted. Here, we will analyse the hourly electricity consumption. Clearly, for business cycle analysis, it may be advantageous to focus on a lower-frequency aggregation level, such as a weekly series, as the volatility of the time series tends to decrease with aggregation level. However, discussing an hourly series will inform our understanding of typical characteristics of higher-frequency time series and thus is relevant for the modelling of daily and weekly series.

As can be seen from Fig. 4, the electricity consumption is characterized by an hour-of-the-week pattern and—especially if we disregard Christmas and fixed holidays—an almost sinusoidal annual seasonality. The latter is termed **autocorrelational seasonality**, as the seasonal impact of each day of the year strongly correlates with the seasonal influences visible in the neighbouring observations. A dependence structure such as this is usually not exploited fully in the seasonal adjustment of lower-frequency time series. Yet, for higher-frequency time series it may improve the estimation of the seasonal effects given the high volatility of the time series and the corresponding estimation uncertainty.

#### Remark 9

Some series show **multilevel periodic effects**. Instead of an hour-of-the-week effect, an hourly series may contain an hour-of-the-day effect and an influence of the day of the week, if the pattern of the intraday-movements remains the same throughout the week and only the level of the series varies from weekday to weekday. By contrast, the magnitude of the intra-day-movements of the electricity consumption changes throughout the week, particularly when contrasting working days and weekends.

The difficulty of estimating all relevant effects is aggravated, as the daily electricity consumption is a relatively **short series**.^7^ As a consequence, we cannot observe all possible calendar and cross-seasonal constellations. To illustrate this point, let us assume that we want to model the interaction between the weekdays and Labour Day. With just over five years of observations, not all possible interactions occurred in the time span considered, and in any case, only very few observations per constellation are included.

At times, higher-frequency time series show rather **uncommon calendar effects**. The daily electricity consumption is impacted by Daylight Saving Time (DST)—even in the hourly series.

The start of the COVID-19 pandemic in Germany again evokes a temporarily declining trend and some slight and gradual changes in the weekly pattern, reflecting the reduction in output in the production sector and possibly changes in the share of people working remotely (for a cross-country comparison of the impact of the pandemic on electricity consumption, see, López Prol and O 2020). For the seasonal adjustment of lower-frequency time series, the pandemic is treated using series of level shifts (LS), additive outliers (AO) and, less frequently, temporary change outliers (TC) with a pre-specified decay rate (Eurostat 2020). The downturn observed for electricity consumption is gradual enough so that one or multiple level shifts are not necessary to model the crisis based on daily data. In general, we observe **non-traditional outlier pattern** more frequently for higher-frequency time series than for monthly or quarterly series. This is neatly illustrated by the finding that the decay rate of 0.7 for TC, the default for monthly series, is often unsuitable for daily or weekly series.

To estimate the hour-of-the-week effect—which encompasses $24 \cdot 7=168$ hours a week—STL is employed with $\gamma _{168}:=7$, i.e. a very short filter that is tailored towards an effect that changes throughout the year. More precisely, the difference between the daily minimum and daily maximum consumption is smaller on the weekend than on working days. This difference is especially small in winter compared to the rest of the year.^8^

After the hour-of-the-week effect has been estimated, the adjusted series aggregated to daily observations serves as input to DSA. We omit the estimation of the day-of-week and day-of-the-month effect, as these effects are not (or no longer) present in this partially adjusted series.

Table 3 shows the estimated moving holiday and cross-seasonal effects for this time series. The cross-seasonal effects included are again interactions between weekday and fixed holidays. As mentioned, due to the length of the series, not all possible calendar interactions can be observed and overall, only very few observations per constellation are available.

**Table 3:** Estimated moving holiday and cross-seasonal effects for German electricity consumption, in percent

Estimate		S.E.	Estimate		S.E.
Carnival Monday	$-1.5$	0.6	Labour Day (bridge)	$-4.8$	1.4
Holy Thursday	$-1.9$	0.7	German Unity (bridge)	$-7.0$	1.0
Good Friday	$-21.6$	0.9	All Saints’ (bridge)	$-4.6$	1.0
Holy Saturday	$-7.4$	0.9	Reformation Day (bridge, after 2017)	$-6.8$	1.5
Easter Sunday	$-6.3$	0.9	National Holidays (Mon-Fri)	$-17.9$	0.4
Easter Monday	$-24.2$	0.9	National Holidays (Sat)	$-4.7$	0.9
Easter Monday (t+1)	$-3.2$	0.7	3d before Christmas (Sat)	1.9	1.1
Ascension (t–1)	$-1.6$	0.7	Christmas Eve (Sat)	4.3	1.8
Ascension	$-22.2$	0.9	Dec 26 (Sat)	5.6	1.2
Ascension (t+1)	$-9.9$	0.7	10d post Dec 26 (Sat)	3.4	0.8
Corpus Christi (t–1)	$-1.1$	0.7	3d before Christmas (Sun)	6.2	1.3
Corpus Christi	$-14.2$	0.9	Christmas Eve (Sun)	8.9	1.6
Corpus Christi (t+1)	$-6.0$	0.7	Christmas Day (Sun)	10.1	1.7
Pentecost (t–1)	1.8	0.6	10d after Dec 26 (Sun)	4.1	0.8
Pentecost	$-23.3$	0.7	Christmas period (Mon-Fri)	$-5.1$	0.7
Pentecost (t+1)	$-3.1$	0.7	Daylight Saving Time Spring	0.5	0.5
			Daylight Saving Time Autumn	$-0.9$	0.6

Based on time series adjusted only for hour-of-the week effects and aggregated to a daily series using daily means. A RegARIMA(5,1,1) model with $2\times 24$ trigonometric terms has been estimated

As discussed above, a noteworthy calendar effect is Daylight Saving Time. DST has an obvious—albeit small—effect on the time series. Here, the moving holiday and cross-seasonal effects will be estimated in the series aggregated to a daily series by averaging the hourly observations. Alternatively, the hourly series could be temporally aggregated to a daily series by summing up the hourly values. Generally, if a multiplicative time series model is used, the difference between the estimated effects using daily averages comparing to those obtained using daily sums is often negligible for calendar constellations that impact the whole day. For example, electricity consumption is estimated to be 23.3 percent lower on Pentecost based on daily averages (see Table 3). If we used daily sums instead, the estimated impact was 23.2 percent.

If we consider matters that affect only single hours of a day, such as DST, the differences can be considerable. Transforming the hourly to a daily series by taking the mean will reduce the estimated impact of DST on the series, because DST will mostly be reduced to capturing the configuration of the hours in a day and the effect on the electricity consumers. If we used hourly sums instead, the estimated impact of DST would additionally include a length-of-the-day effect.

After the calendar and cross-seasonal effects have been estimated, they are broken down into hours, assuming that all hours of the day are influenced in the same way. The hourly series is then adjusted using these hourly factors.

**Fig. 5:**
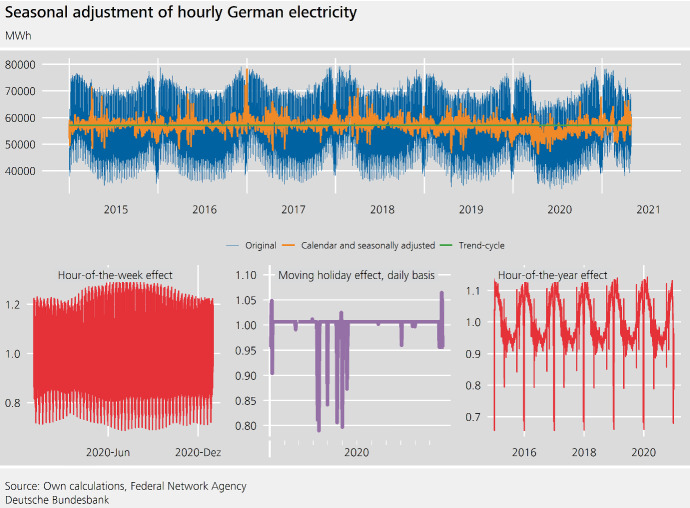
Seasonal adjustment of hourly German electricity

Finally, the hour-of-the-year effect is estimated using STL in DSA with $\gamma _{24\cdot 365}:=13$. The final adjusted series can be seen in Fig. 5.

### Google Trends: Unemployment

Weekly Google Trends data have to be downloaded in chunks of five years. Each of these chunks is a random sample of all search queries and therefore subject to noticeable revisions.^9^ The chunks are chain-linked together and a value of 100 is added to avoid values too close to 0, which can lead to extreme seasonal and calendar factors if a multiplicative model is used.

The series analysed here starts from 10 January 2004 and ends on 10 September 2021. The chunks of the series were downloaded on 13 September 2021.

The definition of the week does not adhere to the ISO 8601 standard. Instead it is defined to start on Sunday and end on Saturday. Such **date and time conventions** are of relevance for regressor construction. For example, Easter Sunday and Easter Monday fall in the same week, and their effect cannot be disentangled. In turn, we only need one regressor that captures the joint effect of Easter Sunday and Easter Monday.

For lower-frequency time series, Christmas and New Year are seasonal effects as they fall in the same period each year. Depending on the modelling strategy, especially if the data are modified so that every year has 365 observations, this holds for daily data too. For weekly time series, as a consequence of their non-isochronicity and date conventions, Christmas’ Day can fall in the 51st or 52nd week of the year, while New Year’s Day falls in week 52, 53 or 1 (ISO 8601 standard) or week 0 or 1 (US convention).

The results of the RegARIMA model estimation are included in Table 4. As discussed, the construction of the data implies that some holidays always fall in the same week. Their impact is therefore indistinguishable. The inclusion of appropriate regressors for the Christmas and New Year’s period can be challenging. Christmas Day always falls in the same week as either Christmas Eve or New Year’s Eve. If both Christmas Eve and New Year’s Eve are included as regressors, then, particularly for short series, separating the impact of Christmas Day from the other two days may be intricate, resulting in an unstable estimation.

**Table 4:** Estimated ARMA coefficients (in absolute terms) and moving holiday effects (in percent) for Google Trends, search term: Arbeitslosigkeit [unemployment]

	Estimate	S.E.		Estimate	S.E.
MA	$-0.87$	0.02	SMA	$-0.75$	0.04
Good Friday	$-5.5$	1.2	Christmas Eve	$-6.7$	1.7
Easter	$-5.4$	1.2	New Year’s Eve	$-5.3$	1.6

A RegARIMA(0,1,1)(0,1,1) model is estimated

Note: The week is defined as starting on Sunday and ending on Saturday. Thus, Easter covers both Sunday and Monday.

**Fig. 6:**
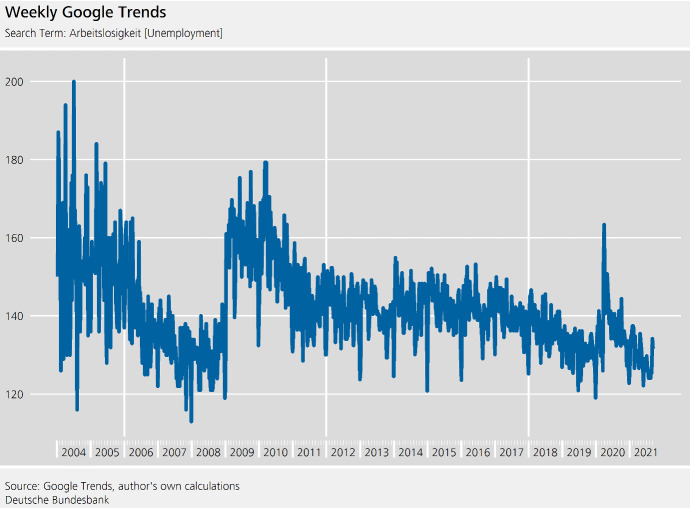
Weekly Google Trends. Search Term: Arbeitslosigkeit (unemployment)

As can be seen from Fig. 6, the series is very volatile, especially at the beginning of the series. It may be difficult to assess the seasonality of the time series visually, but seasonality tests^10^ and the coefficients of the ARIMA model indicate seasonality. To obtain seasonal factors, we rely on the default settings of the fractional airline decomposition.

As the original data are revised considerably every week, the revision policy of this weekly series differs from the daily series discussed above. For the Google Trends series, we follow a partial concurrent adjustment scheme, i.e. the calendar and seasonal components are re-estimated every week, but the order of the RegARIMA model is fixed and is only re-identified annually. The Appendix includes a discussion of graphical tools that can be used for weekly time series if a controlled current adjustment scheme is used.

**Fig. 7:**
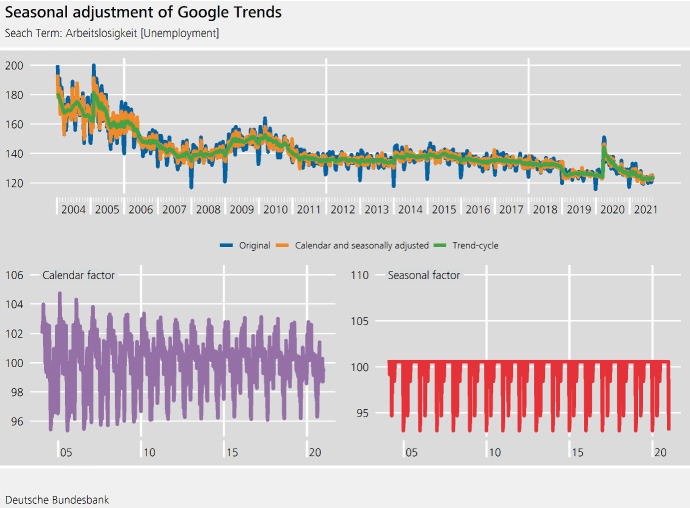
Seasonal adjustment of Google Trends. Search Term: Arbeitslosigkeit (unemployment)

## Summary

The contribution of this paper is twofold. First, we performed an illustrative analysis of a small set of higher-frequency time series. We discussed how these data differ from lower-frequency time series and how this is relevant for seasonal adjustment in general and in light of the COVID-19 pandemic. Second, we developed a taxonomy of the central features of seasonal higher-frequency time series. This list of features can contribute to the assessment of the seasonal adjustment of higher-frequency time series and might serve as a building block in the development of quality diagnostics.

Further research may evaluate different procedures that allow the seasonal adjustment of higher-frequency time series with respect to their ability to handle all of these features.

## Figures and Tables

**Fig. 8:**
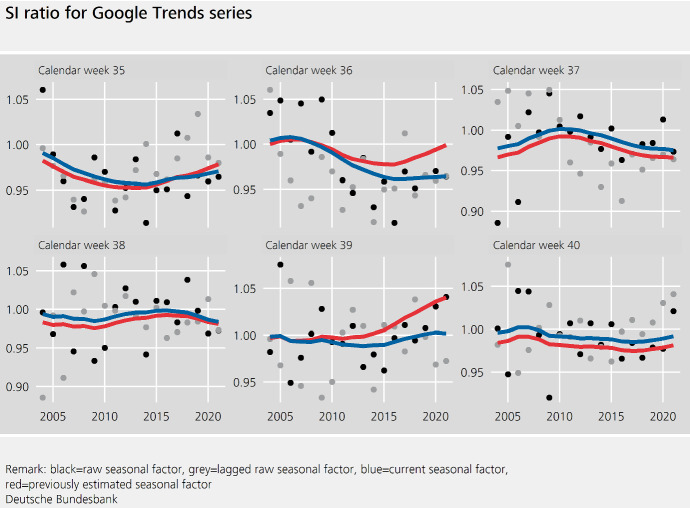
SI ratio for Google Trends series

## A Appendix

For a controlled current adjustment revision scheme, the results from the calendar and outlier adjustment are investigated and, perhaps most importantly, the previously forecasted seasonal components ($\hat{S}^F$) are compared to the new seasonal factors ($\hat{S}^N$) obtained after re-estimation. As reference points for the comparison, the raw seasonal factors ($\hat{S}^{raw}$), i.e. the combined seasonal-irregular component, are included in the analysis. The comparison is either based on tables or graphics, with the latter often being referred to as the SI ratios. The idea is to gauge whether $\hat{S}^N$ better captures the systematic development of $\hat{S}^{raw}$ compared to $\hat{S}^F$. If this is the case to a relevant extent, the previous components are discarded and the re-estimated results are used. Otherwise $\hat{S}^F$ can still be used and the previously published seasonally adjusted values are not revised.

For integer-type periodic influences the construction of the SI ratios is straightforward. Each subplot shows the $\hat{S}^N$, $\hat{S}^{raw}$ and $\hat{S}^F$ for a given period, e.g. a subplot for each month of the year. For series with a non-integer number of observations per year, the configuration is more intricate. For weekly series, the estimated seasonal component of a given observation depends both on observations (multiples of) 52 weeks ago as well as 53 weeks ago and likewise on future observations. Therefore, $\hat{S}^{raw}$ should include all weeks that contribute to the estimation of the seasonal component. For the most recent observations, this is approximately the sequence of weeks with a step size of 52 and the respective previous week. This is shown by way of example in Fig. 8. For any period of the seasonal component, the exact representation of the relevant raw seasonal factors is given by the sequence $(...,\hat{S}^{raw}_{t+\lceil 2\tau \rceil }, \hat{S}^{raw}_{t+\lfloor 2\tau \rfloor }, \hat{S}^{raw}_{t+\lceil \tau \rceil }, \hat{S}^{raw}_{t+\lfloor \tau \rfloor }, \hat{S}^{raw}_t, \hat{S}^{raw}_{t-\lfloor \tau \rfloor }, \hat{S}^{raw}_{t-\lceil \tau \rceil }, \hat{S}^{raw}_{t+\lfloor 2\tau \rfloor }, \hat{S}^{raw}_{t+\lceil 2\tau \rceil }, ...)$.

## Footnotes

1 For a thorough discussion of further decomposition models, see U.S. Census Bureau (2016).

2 The default settings used to capture the trend-cycle have not been changed in the applications presented here; for a discussion of potential parameter settings, we refer to Cleveland et al. (1990)

3 The fractional airline model-based estimation and decomposition are included in the R Package {rjdhf} available on github: https://github.com/palatej/rjdhighfreq. As of version 0.0.4, the Tramo-type estimation of the model does not allow forecasting of the time series. For weekly time series, we therefore use seasonal ARIMA models for pre-processing of the time series with $\tau =52$.

4 Note that Reformation Day - the day before All Saints’ Day—is a regional public holiday. Before 2017, it was only a public holiday in all federal states in eastern Germany. In 2017, Reformation Day it was a national public holiday. From 2018, Reformation Day is a public holiday in the federal states in eastern as well as northern Germany.

5 The spectrum of the series (not shown here) indicates that the series is not influenced by any monthly recurring periodic pattern. Alternatively seasonality tests may be used to check whether there are significant day-of-the-month effects.

6 At the time of writing the ESS Guidelines on Seasonal Adjustment does not discuss this matter and therefore does not stipulate any action (Eurostat, 2015). It could be sensible to define a minimum number of cycles that a new periodic pattern need to pass through before it is regarded as a periodic pattern that needs to be adjusted for.

7 For very short series, it may not be advisable to estimate all seasonal and periodic components. An estimation of the day-of-the-year effect requires at least two years, but for a reliable and sensible estimation usually three or more years of observations are needed. The day-of-the-week effect may often be estimated using less than two years of data.

8 In daily series, this translates into a difference between working days and weekends which increases during the warmer season.

9 The average mean revisions are 7.14 points from week to week in the original series and 6.90 points in the calendar and seasonally adjusted data. The combined seasonal and calendar factor is on average revised by 0.74 percent in absolute terms.

10 We use the seasonality tests recommended by Ollech and Webel (2020) for monthly time series which are implemented in the {seastests} package in R. Setting the frequency of the time series to 52 ignores the non-integer-type seasonality of the series. However, in practice this works well as an approximation. Both the QS and the Friedman test reject the null hypothesis of no seasonality at the 0.1-% level of significance. This also holds true if we only include the last five years of observations.

## References

[CR1] Arora V, Shi S (2016). Energy consumption and economic growth in the United States. Appl Econ.

[CR2] Cleveland RB, Cleveland WS, McRae JE, Terpenning I (1990). STL: a seasonal-trend decomposition procedure based on loess. J Off Stat.

[CR3] Cleveland WS, Devlin SJ (1988). Locally Weighted Regression: An Approach to Regression Analysis by Local Fitting. J Am Stat Assoc.

[CR4] Cleveland WS, Terpenning IJ (1982). Graphical methods for seasonal adjustment. J Am Stat Assoc.

[CR5] De Livera AM, Hyndman RJ, Snyder RD (2011). Forecasting time series with complex seasonal patterns using exponential smoothing. J Am Stat Assoc.

[CR6] Delle Monache D, Emiliozzi S, Nobili A (2020). Tracking economic growth during the Covid-19: a weekly indicator for Italy.

[CR7] Deutsche Bundesbank (2020) Seasonal adjustment of the daily truck toll mileage index. Methodology Report by the Seasonal Adjustment Section

[CR8] Do LPC, Lin K-H, Molnár P (2016). Electricity consumption modelling: a case of Germany. Econ Model.

[CR9] Eraslan S, Götz T (2020) Weekly activity index for the German Economy. Available under https://www.bundesbank.de/wai

[CR10] Eurostat (2020) Guidance on treatment of Covid-19-crisis effects on data

[CR11] Fenz G, Stix H (2021). Monitoring the economy in real time with the weekly OeNB GDP indicator: background.

[CR12] Ladiray D, Palate J, Mazzi GL, Proietti T, Mazzi GL, Ladiray D, Rieser DA (2018). Seasonal Adjustment of Daily and Weekly Data. Handbook on seasonal adjustment, Chapter 29.

[CR13] Lewis DJ, Mertens K, Stock JH (2020) Weekly economic index. https://www.newyorkfed.org/research/policy/weekly-economic-index

[CR14] Lewis DJ, Mertens K, Stock JH, Trivedi M (2020) Measuring real activity using a weekly economic index. Federal Reserve Bank of New York 920

[CR15] Lewis DJ, Mertens K, Stock JH, Trivedi M (2021). High-frequency data and a weekly economic index during the pandemic. In AEA Papers and Proceedings.

[CR16] Lourenço N, Rua A (2021). The daily economic indicator: tracking economic activity daily during the lockdown. Econ Model.

[CR17] López Prol J, O S (2020) Impact of COVID-19 measures on short-term electricity consumption in the most affected EU countries and USA States. iScience 23(10):1–29 10.1016/j.isci.2020.101639 PMC7534594 33043281

[CR18] Ollech D (2018) Seasonal Adjustment of Daily Time Series. Deutsche Bundesbank, Discussion Paper Series 41/2018

[CR19] Ollech D (2021) Seasonal adjustment of daily time series. J Time Ser Econom 13(2): 235–264

[CR20] Ollech D, Gonschorreck N, Hengen L (2021) Flexibilisation of X-11 for Higher-Frequency Data. In: NTTS conference 2021

[CR21] Ollech D, Webel K (2020) A random forest-based approach to identifying the most informative seasonality tests. Discussion Paper No 55/2020, Deutsche Bundesbank

[CR22] Proietti T, Pedregal DJ (2022) Seasonality in high frequency time series. Econom Stat

[CR23] R Core Team (2021) R: A Language and Environment for Statistical Computing. R Foundation for Statistical Computing

[CR24] Svetunkov I (2017) Statistical Models Underlying Functions of ‘smooth’ Package for R. Working paper, Lancaster University Management School

[CR25] U.S. Census Bureau (2016) X-13ARIMA-SEATS reference manual version 1.1. Time Series Research Staff, Statistical Research Division, U.S. Census Bureau

[CR26] Webel K, Alexandria VA (2020). Challenges and recent developments in the seasonal adjustment of daily time series. JSM Proc Bus Econ Stat Sect.

[CR27] Wegmüller P, Glocker C, Guggia V (2021) Weekly economic activity: measurement and informational content. Grundlagen für die Wirtschaftspolitik 17, Staatssekretariat für Wirtschaft SECO

[CR28] Woloszko N (2020) Tracking activity in real time with google trends. OECD Economics Department Working Papers 1634

